# Rapid Destruction of the Humeral Head Caused by Subchondral Insufficiency Fracture: A Report of Two Cases

**DOI:** 10.1155/2015/251696

**Published:** 2015-04-06

**Authors:** Kenichi Goshima, Katsuhiko Kitaoka, Junsuke Nakase, Hiroyuki Tsuchiya

**Affiliations:** ^1^Department of Orthopaedic Surgery, Toyama Municipal Hospital, 2-1 Imaizumi Hokubu-machi, Toyama 939-8511, Japan; ^2^Department of Orthopaedic Surgery, Kijima Hospital, 41-1 Matsudera-machi, Kanazawa 920-0011, Japan; ^3^Department of Orthopaedic Surgery, Graduate School of Medical Science, Kanazawa University, 13-1 Takara-machi, Kanazawa 920-8641, Japan

## Abstract

Rapidly destructive arthritis (RDA) of the shoulder is a rare disease. Here, we report two cases, with different destruction patterns, which were most probably due to subchondral insufficiency fractures (SIFs). Case 1 involved a 77-year-old woman with right shoulder pain. Rapid destruction of both the humeral head and glenoid was seen within 1 month of the onset of shoulder pain. We diagnosed shoulder RDA and performed a hemiarthroplasty. Case 2 involved a 74-year-old woman with left shoulder pain. Humeral head collapse was seen within 5 months of pain onset, without glenoid destruction. Magnetic resonance imaging showed a bone marrow edema pattern with an associated subchondral low-intensity band, typical of SIF. Total shoulder arthroplasty was performed in this case. Shoulder RDA occurs as a result of SIF in elderly women; the progression of the joint destruction is more rapid in cases with SIFs of both the humeral head and the glenoid. Although shoulder RDA is rare, this disease should be included in the differential diagnosis of acute onset shoulder pain in elderly female patients with osteoporosis and persistent joint effusion.

## 1. Introduction

Rapidly destructive arthritis (RDA) is the marked destruction of a joint within months after the onset of symptoms. This condition usually involves the hip [1, 2], with shoulder involvement being rare [3, 4]. Subchondral insufficiency fractures (SIFs) are considered to cause the femoral head collapse associated with hip RDA. However, the pathogenesis of shoulder RDA remains unclear.

We present two cases of shoulder RDA involving different joint destruction patterns, which were probably due to insufficiency fractures. The patients were informed that these data would be submitted for publication, and they consented.

## 2. Case  1

A 77-year-old woman developed right shoulder pain after working on a farm. The symptom did not improve, and she presented to our hospital 1 month after pain onset. The patient did not have a history of trauma, but her medical history included hypertension and osteoporosis. The patient had a 2-year treatment history involving bisphosphonates and the active form of vitamin D. A physical examination revealed right shoulder fluctuation, without local heat or erythema; the active range of motion was 90° in flexion and 10° in external rotation, with an L5 internal rotation level. Her complete blood cell count, erythrocyte sedimentation rate, and serum C-reactive protein levels were normal, and her serum rheumatoid factor was negative. Her synovial fluid was blood-stained but was negative for crystals and infection. A neurologic examination did not reveal evidence of sensory or motor disturbances. Dual-energy X-ray absorptiometry showed reduced bone mineral density (BMD) (68% for the lumbar spine), compared with the young adult mean (YAM).

**Figure 1:**
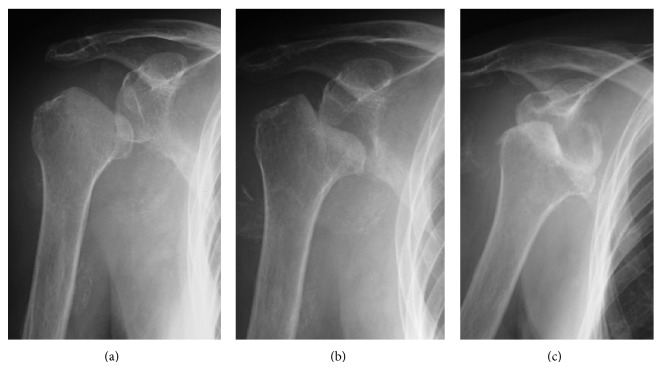
Case  1. Serial preoperative radiographs. (a) At the first visit, the humeral head shows a minor collapse. (b) Three weeks later, the collapse of the humeral head has progressed. (c) Four months later, the destruction of the shoulder is further progressed, including the glenoid.

**Figure 2:**
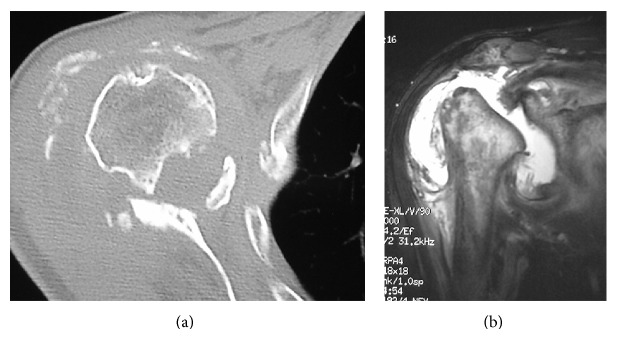
Case  1. Imaging of the right shoulder 4 months after the first visit. (a) An axial computed tomography view demonstrates a destroyed humeral head, displaced bony fragments outside of the greater tuberosity, and destruction of the glenoid. (b) T2-weighted magnetic resonance image (oblique coronal plane) shows the joint destruction, joint effusion, and a rotator cuff tear.

At the patient's first visit, standard radiographs demonstrated some collapse of the humeral head (Figure 1(a)) and had progressed during the following 3 weeks (Figure 1(b)). Radiographs taken 4 months later also revealed destruction of the glenoid (Figure 1(c)). Computed tomography (CT) indicated a destroyed humeral head, displaced bony fragments outside of the greater tuberosity, and destruction of the glenoid (Figure 2(a)). Magnetic resonance imaging (MRI) revealed joint effusion in the glenohumeral joint and a rotator cuff tear, but tumor masses and synovial hyperplasia were not observed (Figure 2(b)).

Neuropathic arthropathy and infectious arthritis were excluded, based on clinical and laboratory findings. Therefore, we performed a hemiarthroplasty. A histologic examination revealed abundant new bone formation but no evidence of osteonecrosis in the subchondral area. One year postoperatively, the patient was pain-free, without daily life activity restrictions.

## 3. Case  2

**Figure 3:**
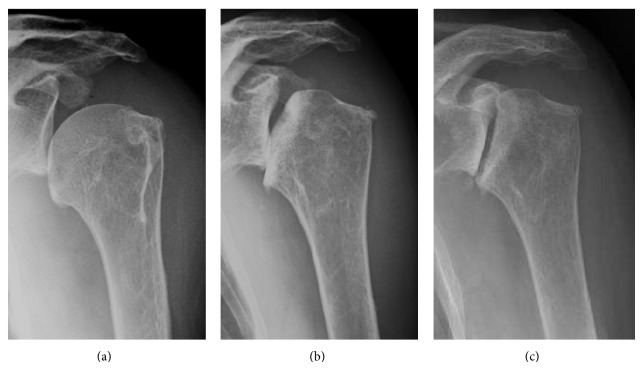
Case  2. Serial preoperative radiographs. (a) The humeral head is slightly displaced inferiorly, but the shape is maintained at the time of symptom onset. (b) Five months later, the humeral head demonstrates progressed collapse. (c) Bone defects of the glenoid are noted 2 years after symptom onset.

A 74-year-old woman presented to our hospital with a 2-year history of left shoulder pain and persistent joint effusion. She had previously visited a nearby hospital and had been conservatively treated with intra-articular injection of a mixture of local anesthetic and corticosteroid. Radiography of her left shoulder at that time looked normal (Figure 3(a)). However, some collapse of the humeral head was found 5 months after symptom onset (Figure 3(b)). Despite the radiological findings, she desired conservative treatment. However, her pain persisted, even at night, and she was referred to our hospital for further treatment, 2 years after symptom onset. A physical examination conducted at our hospital revealed that her left shoulder was not swollen, with an active range of motion of 170° in flexion and 10° in external rotation; the internal rotation level was at L5. Neurological abnormalities were not noted and her blood test results did not show evidence of infectious disease or rheumatoid arthritis. Her synovial fluid was blood-stained but negative for crystals or infection. Additionally, her lumbar spine BMD (%YAM) was 67%.

**Figure 4:**
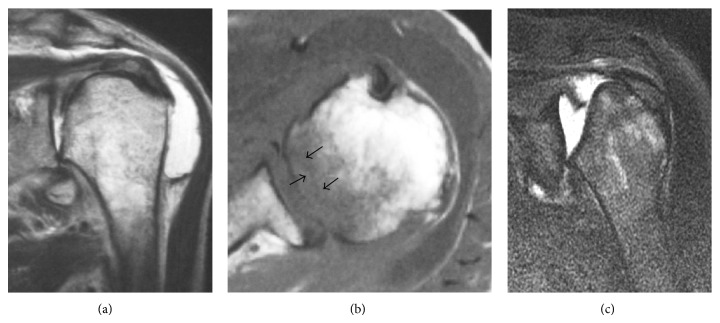
Case  2. Magnetic resonance image (MRI) of the left shoulder at symptom onset (a, b) and at the first visit to our hospital (c). (a) T2-weighted MRI (oblique coronal plane) demonstrates joint effusion and a slightly collapsed humeral head. (b) Axial, T1-weighted MRI shows a subchondral serpiginous pattern of low signal intensity with associated bone marrow edema. (c) T2-weighted MRI (coronal view) demonstrates the collapse of the humeral head and an intact rotator cuff.

Radiography demonstrated that the humeral head was slightly displaced inferiorly, but its shape was maintained at the time of symptom onset (Figure 3(a)). Subsequent radiographs, taken 5 months later, revealed collapse of the humeral head (Figure 3(b)). Upon the patient's first visit to our hospital, 2 years after symptom onset, the radiographs demonstrated bone defects of the glenoid (Figure 3(c)). At symptom onset, oblique coronal MRI showed joint effusion and slight collapse of the humeral head (Figure 4(a)). An axial T1-weighted MRI showed a subchondral serpiginous pattern of low signal intensity with associated bone marrow edema (Figure 4(b)). At our hospital, oblique coronal MRI demonstrated the collapse of the humeral head, but the rotator cuff remained intact. Tumors, synovial hyperplasia, and intraosseous cystic lesions were not demonstrated (Figure 4(c)).

**Figure 5:**
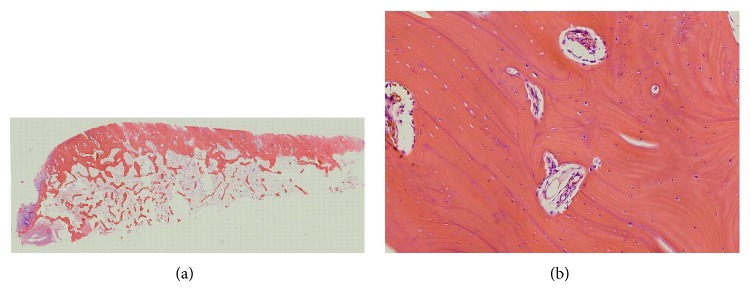
Histologic features of the excised humeral head in patient 2 (hematoxylin-eosin staining). (a) A coronal section of the humeral head exhibits the collapse of the subchondral area and articular cartilage defects (×1.25). (b) The bone trabeculae are mostly vital but focally necrotic. New bone formation is seen around the necrotic bone trabeculae (×100).

A total shoulder arthroplasty was carried out because the rotator cuff was intact. The excised humeral head had detached articular cartilage and collapsed subchondral bone. Histologically, the articular cartilage was lost. In the subchondral area, the bone trabeculae were mostly vital but were focally necrotic. New bone formation was seen around the necrotic bone trabeculae (Figure 5).

At the 4-year follow-up, the patient was free of symptoms, with an active range of motion of 170° in flexion and 70° in external rotation; the internal rotation level was L5. Additionally, her shoulder prosthesis had not failed, and further progressive bone destruction had not occurred in her left shoulder.

## 4. Discussion

RDA of the shoulder was described first by Smith and Adams in the nineteenth century [7]. Later, Nguyen [4] reviewed 60 cases of shoulder RDA and concluded that most cases occurred in elderly women and that large amounts of synovial fluid characterized this condition. The 2 cases reported here showed characteristics similar to those previously described.

Radiographically, RDA may mimic other destructive diseases of the shoulder, including neuropathic arthropathy, crystal-induced arthritis, dialysis arthropathy, neoplasm, rheumatoid arthritis, pyogenic arthritis, and avascular osteonecrosis of the humeral head. In our cases, the first six of these diseases were excluded by clinical findings, including each patient's medical history, blood test results, and synovial fluid findings. In addition, avascular osteonecrosis of the humeral head was excluded on the basis of the histological findings.

The pathogenesis of shoulder RDA has not been identified. However, Yamamoto et al. [8–10] proposed that SIF, resulting from osteopenia, might lead to RDA of the hip. They reported that MRI findings are characterized by a subchondral, linear, serpiginous pattern of very low signal intensity on the T1-weighted images and an associated bone marrow edema pattern. The subchondral low-intensity band was histologically shown to be a fracture line. In our case 2, MRI showed these typical findings. Recently, Yoshikawa et al. [6] and Tokuya et al. [5] reported that the cause of shoulder RDA was SIF due to bone fragility, as previously reported for the hip joint. In the present study, both of our cases showed decreased BMD (osteoporosis). Furthermore, in case 2, MRI showed a bone marrow edema pattern with an associated subchondral low-intensity band on the T1-weighted image, which is typical of SIF. Therefore, we presumed that the shoulder RDA was caused by the SIF due to bone fragility, as previously reported.

Our cases showed different patterns of progressive shoulder destruction. In case 1, rapid destruction of both the humeral head and glenoid was seen within 1 month after symptom onset. In case 2, collapse of the humeral head was seen within 5 months, without glenoid destruction. Motomura et al. [11] have suggested that SIF of the femoral head and acetabulum may produce more excessive load on the cartilage, leading to rapid destruction of the hip joint, compared with the destruction associated with an isolated femoral head lesion. Yoshikawa et al. [6] reported 2 RDA cases without SIF of the glenoid. In their report, the times to humeral head collapse were 3 or 8 months (Table 1). Meanwhile, Tokuya et al. [5] reported a case of RDA, with SIF of the glenoid, in which humeral head collapse occurred within 1 month after the first visit. Therefore, we presume that SIF was present in both the humeral head and glenoid in case 1. In addition, both case 1 and the patient reported by Tokuya et al. [5] had massive rotator cuff tears, which might have led to shoulder joint instability and subsequent rapid destruction.

## 5. Conclusion

We presented two cases of shoulder RDA demonstrating different destruction patterns. Rapid shoulder destruction occurs as a result of SIFs in elderly women, and the joint destruction is more rapid in patients with SIFs of both the humeral head and glenoid. Although shoulder RDA is a rare condition, this disease should be included in the differential diagnosis of acute onset shoulder pain in elderly female patients with osteoporosis and persistent joint effusion.

## Figures and Tables

**Table 1:** Literature review of shoulder rapidly destructive arthritis.

Author	Age (years)	Sex	Osteoporosis	Time to humeral head collapse (months)	Rotator cuff tear	Glenoid subchondral insufficiency fracture
Tokuya et al. [5]	77	Female	+	1	+	+

Yoshikawa et al. [6]	74	Female	+	8	−	−
	78	Female	+	3	−	−
